# Fingolimod Effects on Motor Function and BDNF-TrkB Signaling in a Huntington’s Mouse Model Are Disease-Stage-Dependent

**DOI:** 10.3390/ijms27010494

**Published:** 2026-01-03

**Authors:** Khanh Q. Nguyen, Vladimir V. Rymar, Abbas F. Sadikot

**Affiliations:** Cone Laboratory, Department of Neurology & Neurosurgery, Montreal Neurological Institute, McGill University, Montreal, QC H3A 2B4, Canada; khanh.nguyen@mail.mcgill.ca (K.Q.N.); vladimir.rymar@mail.mcgill.ca (V.V.R.)

**Keywords:** neurotrophin, brain-derived neurotrophic factor, sphingosine-1-phosphate, R6/2 mouse, neurodegeneration, neuroprotection, striatum, cerebral cortex, TrkB, motor behavior

## Abstract

Huntington’s Disease (HD) is characterized by prominent degeneration of the principal neurons of the striatum and by progressive motor and cognitive deterioration. Striatal neurons degenerate in HD due to multiple cell-autonomous and non-autonomous factors. Impaired neurotrophin signaling by brain-derived neurotrophic factor (BDNF) and its cognate receptor Tropomyosin receptor kinase B (TrkB) is an important mechanism underlying neuronal loss in HD. Fingolimod, a clinically approved oral drug for Multiple Sclerosis, was originally developed based on its anti-inflammatory properties. Recent work suggests that fingolimod can also promote BDNF expression and enhance neurotrophic support in the brain. We hypothesized that fingolimod treatment initiated during the presymptomatic phase would increase striatal BDNF levels and protect against motor dysfunction in HD. In wild-type mice, fingolimod treatment increases striatal BDNF levels and enhances BDNF-TrkB signaling. However, chronic fingolimod therapy (0.1 mg/kg, i.p., twice per week, over 7 weeks) initiated at age 4 weeks in the R6/2 mouse model of HD failed to improve behavioral locomotor deficits and exacerbated limb clasping. Furthermore, fingolimod treatment in these presymptomatic R6/2 mice acutely decreased BDNF-TrkB signaling in the striatum in a dose-dependent manner. In contrast, acute administration of fingolimod in symptomatic 7-week-old R6/2 mice increased striatal BDNF-TrkB signaling in a dose-dependent manner, consistent with previous work suggesting that chronic fingolimod can improve motor behavior when given during the symptomatic phase. Thus, the effects of fingolimod striatal BDNF-TrkB signaling and motor behavior in HD are complex and vary with disease stage. Addressing this variability is critical for the design of neuroprotective drug trials in HD, including those utilizing sphingosine-1-phosphate receptor (S1P) modulators.

## 1. Introduction

The striatum, a central nucleus within the basal ganglia, plays a pivotal role in processing programs that shape motor, cognitive, and emotional behaviors [1,2,3]. GABAergic medium spiny projection neurons (MSPs) constitute the majority (>90%) of striatal neurons, with a smaller population of interneurons. Striatal MSPs express TrkB (Tropomyosin receptor kinase B), the receptor for brain-derived neurotrophic factor (BDNF). MSPs rely on the BDNF-TrkB neurotrophic signaling pathway to promote developmental survival [4,5], maintain MSP phenotype [6,7,8], modulate neurotransmitter activity [9,10], and protect against toxic stressors [8,11,12]. For example, BDNF-TrkB signaling is essential for maintaining the expression of DARPP-32, an MSP-enriched protein that is a central regulator of neurotransmitter signaling in MSPs [13,14,15].

Early dysfunction and prominent degeneration of striatal MSPs are key factors contributing to the hallmark hyperkinetic motor symptoms of Huntington’s disease (HD) [16,17,18]. HD is an autosomal dominant genetic disorder caused by polymorphic expansions of CAG trinucleotide repeats in the *HTT* gene, which produces mutant huntingtin protein with an elongated N-terminal poly-glutamine domain [19]. The expression of mutant huntingtin protein leads to the disruption of various cellular processes that increase the vulnerability of striatal neurons to degeneration and drive pathological progression [20,21]. Reduced afferent BDNF supply [22,23,24,25] and impaired TrkB signaling [26,27] are implicated in the degeneration of striatal MSPs in HD. Therefore, enhancing BDNF-TrkB signaling may be a promising therapeutic strategy for mitigating striatal degeneration and improving motor and cognitive symptoms in HD [28,29].

Fingolimod, a clinically approved oral drug for Multiple Sclerosis [30], is primarily known for its ability to reduce pathogenic immune responses [31]. However, recent work suggests that it may also offer neuroprotective effects by enhancing neurotrophic support in various animal models of neurodegenerative diseases [32,33,34,35,36]. Fingolimod is lipophilic and readily crosses the blood–brain barrier to exert direct effects on cells in the central nervous system (CNS) [37,38,39]. It is converted to phospho-fingolimod (p-fingolimod), an analogue of sphingosine-1-phosphate (S1P), a naturally occurring signaling molecule that activates various S1P receptors (S1P1-5 subtypes). P-fingolimod and S1P can bind S1P receptors that modulate glutamate release and excitatory activity in neurons [40,41,42,43]. Additionally, p-fingolimod can act as a functional antagonist by inducing S1P receptor internalization [44,45], potentially attenuating endogenous S1P-mediated effects on neuronal activation [46]. Given its complex pharmacodynamics and potential therapeutic role in HD, it is crucial to evaluate fingolimod’s effects, particularly during the prodromal phase prior to neuronal degeneration, before considering broader clinical applications [47,48]. Notably, fingolimod can promote activity-induced expression of BDNF, enhancing neurotrophic support for neurons [32,49]. Moreover, in a mouse model of Rett’s syndrome, early therapy with fingolimod attenuates motor symptoms in association with enhanced BDNF-mediated striatal neuroprotection [32]. We therefore investigated the effectiveness of initiating fingolimod therapy at a presymptomatic stage in a mouse model of HD.

We employed the R6/2 transgenic mouse model of HD, which expresses the N-terminal fragment of mutant huntingtin with approximately 120 glutamine repeats. By 9 weeks of age, this model displays significant motor dysfunction, with progressive decrease in spontaneous locomotion, impaired coordination on a Rotarod test, and limb-clasping dystonia. These motor deficits coincide with the progressive degeneration of striatal MSPs [50,51,52]. The R6/2 model has been extensively used to investigate neurotrophic mechanisms contributing to striatal neuron vulnerability in HD, including impaired BDNF-TrkB signaling [27,53,54,55]. We hypothesized that early intervention with chronic fingolimod therapy could protect R6/2 mice from motor decline by increasing BDNF levels and enhancing TrkB signaling in the striatum. However, our results demonstrate that chronic fingolimod treatment, when initiated in young presymptomatic R6/2 mice, does not improve progressive decline in motor function at later stages. Moreover, we observed a decrease in striatal BDNF-TrkB signaling in these presymptomatic R6/2 mice after a single dose of fingolimod. In contrast, acute fingolimod administration in older symptomatic mice led to a significant increase in striatal BDNF-TrkB signaling. These results indicate that the effects of S1P receptor ligands such as fingolimod depend on the neurodegenerative state. Therapeutic effects may therefore vary depending on whether the drug is administered during presymptomatic or later phases of HD.

## 2. Results

### 2.1. Effects of Chronic Fingolimod on Motor Behavior in WT and R6/2 Mice

Motor phenotypes in R6/2 mice are noted mainly after age 7 weeks, with progressive worsening of locomotor activity, incoordination, dystonic phenotype, and associated reduction in weight gain [52,56,57]. Cohorts of WT mice and R6/2 littermates were divided into two treatment groups receiving either saline or fingolimod (0.1 mg/kg, i.p., every 3.5 days- q3.5d) starting at 4 weeks of age, prior to onset of R6/2 motor symptoms. Motor behavior was assessed at regular intervals up to 11 weeks. This dosing regimen was based on previous work in a mouse model of Rett’s syndrome, where fingolimod (0.1 mg/kg i.p.) administration every 4 days for 2–4 weeks improved motor behavior and increased striatal and cortical BDNF levels [32].

**Figure 1:**
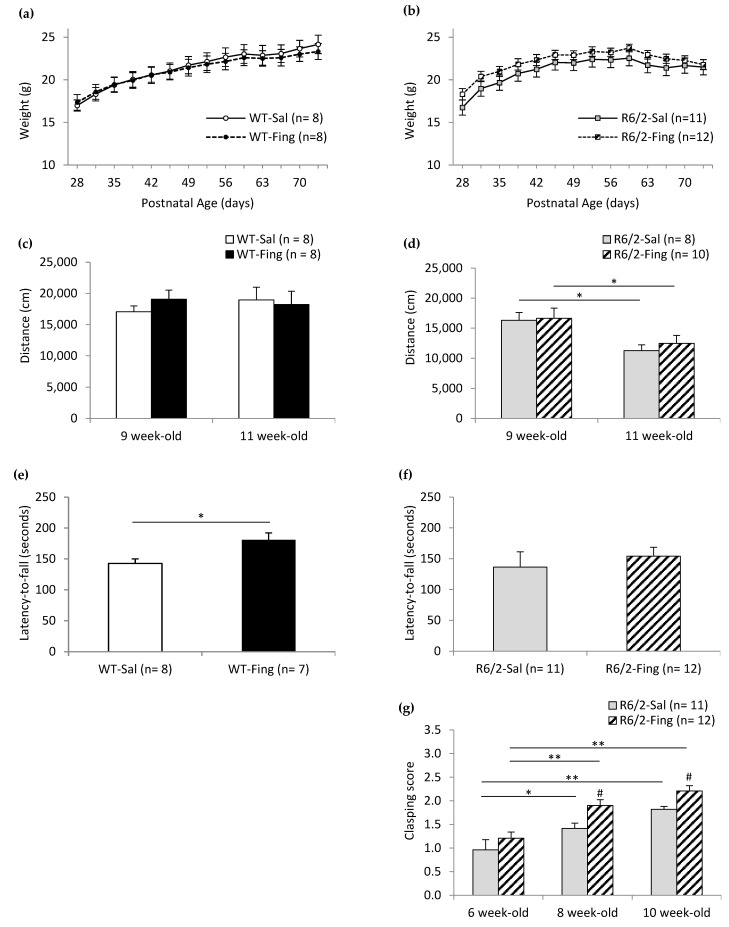
Effects of chronic fingolimod treatment on locomotor behavior. WT (wildtype) and R6/2 mice received saline (i.p., q3.5d; Sal) or fingolimod (0.1 mg/kg, i.p., q3.5d; Fing) treatments starting at age 4 weeks. (**a**) WT mice show progressive weight gain in both saline and fingolimod treatment groups. (**b**) R6/2 mice show impaired weight gain after age 7 weeks in both treatment groups. (**c**) Open field assay shows fingolimod does not alter spontaneous locomotor activity in either WT or (**d**) R6/2 mice, and R6/2 mice exhibited age-related decline in locomotor activity regardless of treatment, reflecting progression of the degenerative process (* *p* < 0.050). (**e**) Rotarod assay shows that fingolimod improves motor coordination in 10-week-old WT mice, as indicated by the increased latency to fall (* *p* < 0.050). (**f**) However, no such effect was observed in R6/2 mice with similar latency to fall for both treatment groups. (**g**) Limb clasping scores increase with age in R6/2 mice, indicating progressive motor dystonia in both treatment groups (* *p* < 0.050; ** *p* < 0.001). Fingolimod treatment exacerbates limb dystonia in R6/2 mice at both 8 and 10 weeks of age (# *p* < 0.050 vs. saline).

To determine the effect of fingolimod on weight gain, WT and R6/2 mice in saline or drug treatment groups were weighed twice per week from 4 to 11 weeks of age (Figure 1a,b). Both WT and R6/2 mice exhibited age-related changes in weight, with comparatively less weight gain in the HD model. Fingolimod treatment mildly impaired weight gain over time in WT mice but did not affect weight in R6/2 mice. WT mice receiving fingolimod showed reduced weight gain compared to saline controls after 8.5 weeks of age. In contrast, both saline- and fingolimod-treated R6/2 mice displayed similar weight gain patterns, peaking at 8.5 weeks before declining. Specifically, statistical analysis (two-factor mixed ANOVA) of weight data for WT mice (Figure 1a) indicates significant effects of Age (F_13,182_ = 228.726; *p* < 0.001) and interaction of Age x Treatment (F_13,182_ = 1.788; *p* < 0.050) but no effect of Treatment (F_1,14_ = 0.033; *p* = 0.858), suggesting that fingolimod-treated WT mice exhibit a different age-related weight gain compared to saline-treated WT mice. Post hoc (Bonferroni) comparisons within the WT saline group (*n* = 8) show higher week-over-week weights up to 10.5 weeks (e.g., 9.5 vs. 10.5 weeks: 23.1 ± 1.0 g vs. 24.2 ± 1.0 g; *p* < 0.050). Post hoc comparisons within the WT fingolimod group (*n* = 8) show higher week-over-week weights only up to 8.5 weeks (e.g., 7.5 vs. 8.5 weeks: 21.8 ± 1.0 g vs. 22.6 ± 1.0 g; *p* < 0.050). In R6/2 mice (Figure 1b), statistical analysis of weights indicates a significant effect of Age (F_13,273_ = 75.472; *p* < 0.001) but no effect of Treatment (F_1,21_ = 0.820; *p* = 0.376). Post hoc comparisons within the R6/2 saline group (*n* = 11) show higher week-over-week weights up to 7 weeks (e.g., 6 vs. 7 weeks: 21.2 ± 0.9 g vs. 22.0 ± 0.8 g; *p* < 0.001) with a peak at 8.5 weeks (22.5 ± 0.9 g), followed by a lack of weight gain in older mice (e.g., 10.5 weeks: 21.5 ± 0.8 g; *p* = 0.964 vs. 8.5 weeks). Post hoc comparisons within the R6/2 fingolimod group (*n* = 12) show weights are higher week-over-week up to age 6.5 weeks (e.g., 5.5 vs. 6.5 weeks: 21.8 ± 0.9 g vs. 22.9 ± 0.8 g; *p* < 0.050) and then peak at 8.5 weeks (23.7 ± 0.9 g) and become significantly lower at older ages (e.g., 10.5 weeks: 21.8 ± 0.8 g; *p* < 0.050 vs. 8.5 weeks).

Open field assessments at 9 and 11 weeks revealed no significant effect of fingolimod on spontaneous locomotor activity in either WT or R6/2 mice (Figure 1c,d). While R6/2 mice exhibited a significant age-related decline in locomotor activity (F_1,16_ = 43.612; *p* < 0.001), the decline was similar in both saline and fingolimod treatment groups, indicating that fingolimod does not alter the natural progression of locomotor deficits in this HD model.

To determine the effects of fingolimod on motor coordination and balance, WT and R6/2 mice were assessed at age 10 weeks on an Accelerating Rotarod apparatus (Figure 1e,f). WT mice performed better with fingolimod treatment (181 ± 11 s, *n* = 7; *p* < 0.050) compared to saline treatment (143 ± 7 s, *n* = 8). R6/2 mice performed similarly with fingolimod treatment (154 ± 14 s, *n* = 12) compared to saline treatment (136 ± 25 s, *n* = 11).

To determine if fingolimod treatment alters dystonic behavior in R6/2 mice, limb clasping during tail suspension was assessed in saline- and fingolimod-treated groups at age 6, 8, and 10 weeks (Figure 1g). ANOVA of clasping scores shows significant effects of Age (F_2,42_ = 44.082; *p* < 0.001) and Treatment (F_1,21_ = 6.028; *p* < 0.050). Post hoc (Bonferroni) comparisons within the saline group show higher clasping scores at 8 weeks (1.42 ± 0.12, *n* = 11; *p* < 0.050) and 10 weeks (1.82 ± 0.10, *n* = 11; *p* < 0.001) compared to 6 weeks (0.96 ± 0.18, *n* = 11). Post hoc comparisons within the fingolimod group show higher clasping scores at 8 weeks (1.90 ± 0.12, *n* = 12; *p* < 0.001) and 10 weeks (2.21 ± 0.09, *n* = 12; *p* < 0.001) compared to 6 weeks (1.21 ± 0.17, *n* = 12). Post hoc comparisons between treatment groups show lower clasping scores in the saline vs. fingolimod group (8 weeks: 1.42 + 0.12 vs. 1.90 + 0.12; *p* < 0.050; 10 weeks: 1.82 ± 0.10 vs. 2.21 ± 0.09; *p* < 0.050). Thus, chronic fingolimod treatment when initiated in presymptomatic R6/2 mice exacerbates limb clasping dystonia.

### 2.2. Effects of Chronic Fingolimod on BDNF-TrkB Signaling in Forebrain Motor Regions of R6/2 Mice

Given that increased striatal BDNF-TrkB signaling has been linked to improvements in motor and learning deficits in various neurodegenerative disease models [32,33,58], we investigated whether this protein pathway is altered in striatal tissue samples from the above cohort of R6/2 mice at the end of behavioral testing, following chronic fingolimod treatment (0.1 mg/kg, i.p., q3.5d) from 4 to 11 weeks of age. Motor cortex samples were also analyzed for comparison.

**Figure 2:**
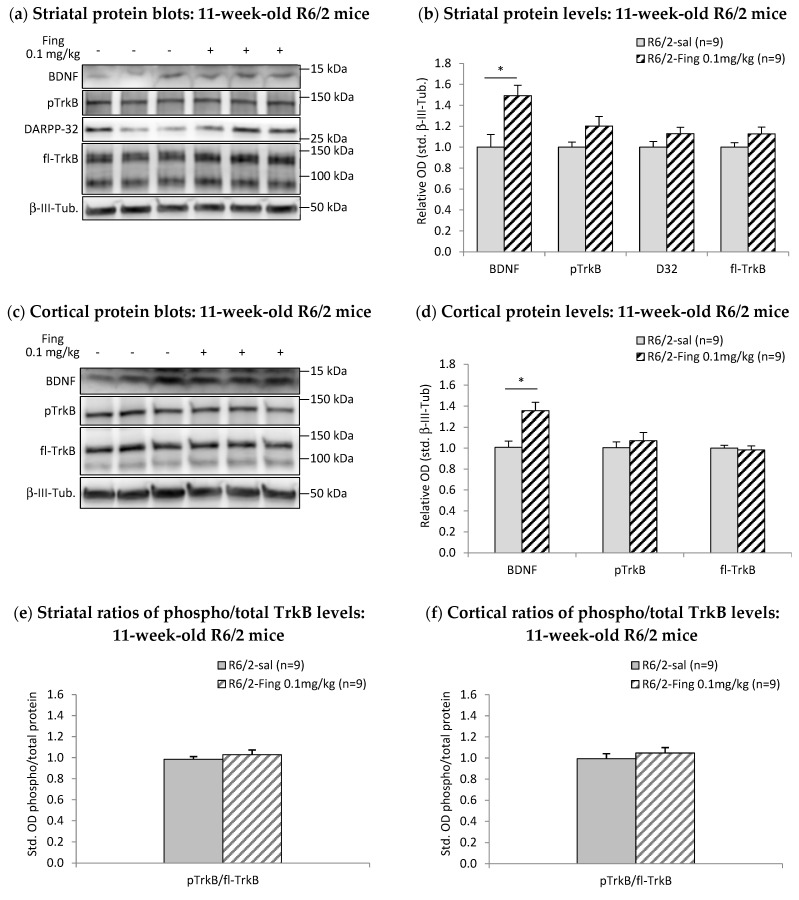
Effects of chronic fingolimod treatment on BDNF-TrkB signaling proteins in forebrain of 11-week-old R6/2 mice. (**a**) Representative Western blot of striatal proteins (rows): BDNF (14 kDa), phospho-(p)TrkB (145 kDa), DARPP-32 (32 kDa), full-length (fl)TrkB (145 kDa), and β-III-Tubulin (50 kDa) from individual (columns) R6/2 mice after chronic treatment with saline (sal, −) or fingolimod (0.1 mg/kg, i.p.; Fing, +). Molecular weight standards are indicated on the right. (**b**) Graph of relative levels of striatal BDNF and downstream signaling proteins in R6/2 mice normalized to saline controls. Striatal BDNF levels are higher after chronic fingolimod treatments vs. saline controls (* *p* < 0.050). (**c**) Representative blots of motor cortex proteins from individual R6/2 mice after chronic treatment with saline (−) or fingolimod (+). (**d**) Graph of relative cortical protein levels in R6/2 mice shows that BDNF levels are higher after chronic fingolimod treatments vs. saline controls (* *p* < 0.050). (**e**) Ratios of pTrkB/total TrkB levels in the striatum and (**f**) motor cortex in 11-week-old R6/2 mice show that the activation of TrkB signaling is similar after chronic fingolimod treatments vs. saline controls.

Striatal protein extracts from individual fingolimod- or saline-treated 11-week-old R6/2 mice were run in parallel on Western Blots (Figure 2). Optical density analysis of immunoblot images of BDNF-TrkB pathway proteins [7,27,59,60] was used to calculate relative protein values standardized to the saline-treated R6/2 group. The results show striatal BDNF levels are significantly higher in fingolimod-treated R6/2 mice (relative optical density—rel. OD = 1.49 ± 0.10, *n* = 9) compared to saline controls (rel. OD = 1.00 ± 0.12, *n* = 9; *p* < 0.050) (Figure 2a,b). Total levels of the downstream pathway proteins, full-length TrkB receptors, and DARPP-32 are similar between the two treatment groups (Figure 2a,b). Levels of activated phospho-TrkB receptors are marginally higher in fingolimod-treated R6/2 mice (fingolimod: rel. OD = 1.20 ± 0.09, *n* = 9; saline: OD = 1.00 ± 0.05, *n* = 9; *p* = 0.071). Additional analyses of the ratio of activated phospho-TrkB relative to total TrkB receptors in the striatum show that TrkB signaling activation in chronic fingolimod-treated R6/2 mice (1.03 ± 0.03, *n* = 9) is similar to saline controls (0.98 ± 0.05, *n* = 9) (Figure 2e). 

BDNF levels are also higher in the motor cortex of fingolimod-treated 11-week-old R6/2 mice (rel. OD = 1.36 ± 0.08, *n* = 9) compared to saline controls (rel. OD = 1.01 ± 0.06, *n* = 9; *p* < 0.050) (Figure 2c,d). Cortical levels of activated phospho-TrkB and total full-length TrkB receptors are not altered by chronic fingolimod treatment (Figure 2c,d). The ratio of activated phospho-TrkB–total TrkB in the cortex of chronic fingolimod-treated R6/2 mice (fingolimod: 1.05 ± 0.06, *n* = 9) is similar to saline controls (0.99 ± 0.05, *n* = 9) (Figure 2f).

In summary, chronic treatment with fingolimod (0.1 mg/kg) initiated in 4-week-old R6/2 mice augments BDNF levels in the cortex and striatum by 11 weeks but does not enhance downstream TrkB signaling or DARPP-32 levels in the striatum.

### 2.3. Acute Effects of Fingolimod on BDNF-TrkB Signaling in Presymptomatic and Symptomatic R6/2 Mice

**Figure 3:**
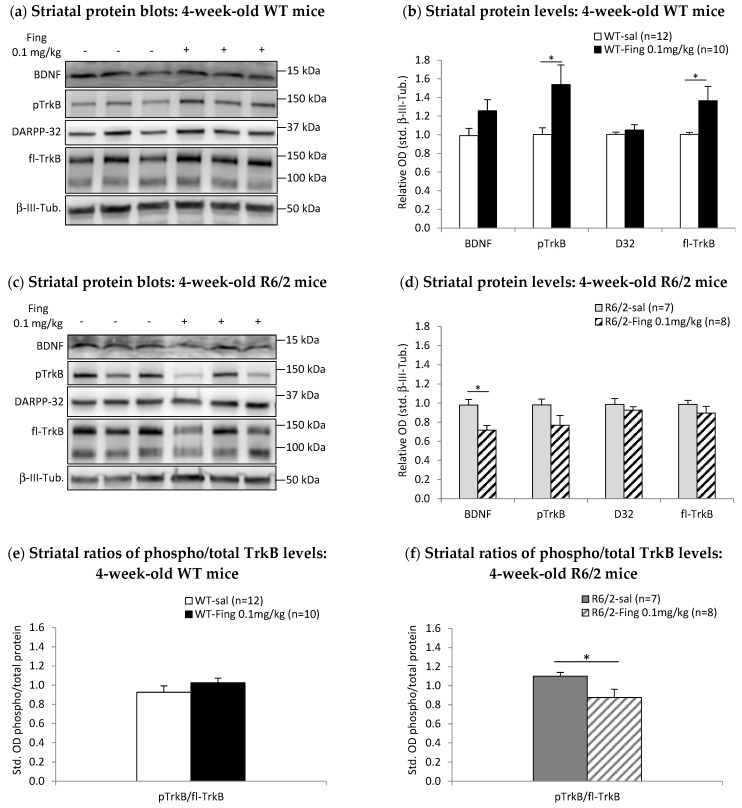
Effects of acute fingolimod treatment on BDNF-TrkB signaling proteins in 4-week-old WT and R6/2 mice. At 48 h after a single injection of saline (sal) or 0.1 mg/kg fingolimod (Fing), striatal tissues were extracted and analyzed by Western blot. (**a**) Representative blots of striatal proteins (rows) from individual (columns) WT mice treated at 4 weeks with saline (−) or fingolimod (+). (**b**) Graph of relative levels of striatal BDNF and downstream signaling proteins in WT mice normalized to saline controls. pTrkB and fl-TrkB levels are increased by this low dose of fingolimod vs. saline controls at 4 weeks (* *p* < 0.050). (**c**) Representative blots of striatal proteins from individual R6/2 mice treated at 4 weeks with saline (−) or fingolimod (+). (**d**) Graph of relative levels of striatal proteins in R6/2 mice shows that BDNF, pTrkB, and fl-TrkB are decreased by this low dose of fingolimod vs. saline controls at 4 weeks (* *p* < 0.050 ). (**e**) Ratios of activated pTrkB/total TrkB levels in the striatum of WT mice and (**f**) R6/2 mice show that activation of TrkB signaling is attenuated after fingolimod treatment in R6/2 mice (* *p* < 0.050 vs. saline).

Previous work indicates that initiating chronic fingolimod therapy in symptomatic R6/2 mice (i.e., from 7- or 10-week-old) improves some motor deficits and correlates with increases in striatal BDNF protein levels [35]. In contrast, we find that initiating chronic fingolimod therapy in presymptomatic 4-week-old R6/2 mice does not improve spontaneous locomotion in an open field and worsens limb clasping symptoms. We therefore determined if fingolimod has distinct acute effects on striatal BDNF-TrkB signaling in presymptomatic and symptomatic R6/2 mice. Striatal tissue was extracted 48 h after a single injection of fingolimod at the 0.1 mg/kg dose used for chronic therapy. BDNF-TrkB signaling protein levels were compared between fingolimod- vs. saline-treated WT and R6/2 mice (Figure 3).

BDNF levels are higher in the striatum of 4-week-old WT mice 48 h after a single dose of 0.1 mg/kg fingolimod (rel. OD = 1.26 ± 0.12, *n* = 10) compared to saline controls (rel. OD = 0.99 ± 0.08, *n* = 12) (Figure 3a,b), but the difference is only marginally significant (*p* = 0.067). However, striatal levels of downstream activated phospho-TrkB receptors are significantly higher in the fingolimod group (rel. OD = 1.54 ± 0.21, *n* = 10; *p* < 0.050) compared to saline controls (rel. OD = 1.00 ± 0.07, *n* = 12). Levels of total full-length TrkB receptors are also significantly higher in the fingolimod group (rel. OD = 1.36 ± 0.15, *n* = 10; *p* < 0.050) compared to saline controls (rel. OD = 1.00 ± 0.02, *n* = 12). Striatal levels of DARPP-32 remain unchanged with 0.1 mg/kg fingolimod treatment in 4-week-old WT mice (Figure 3a,b). The ratio of activated phospho-TrkB–total TrkB in the striatum is similar between fingolimod- compared to saline-treated 4-week-old WT mice (fingolimod: 1.03 ± 0.05, *n* = 10; saline: 0.93 ± 0.07, *n* = 12) (Figure 3e).

In contrast to WT mice, striatal BDNF levels are significantly lower in 4-week-old R6/2 mice after acute treatment with 0.1 mg/kg fingolimod (rel. OD = 0.72 ± 0.05, *n* = 8; *p* < 0.050) compared to saline controls (rel. OD = 0.98 ± 0.06, *n* = 7) (Figure 3c,d). Striatal phospho-TrkB levels are also lower in the fingolimod group (rel. OD = 0.77 ± 0.10, *n* = 8) compared to saline controls (rel. OD = 0.98 ± 0.06, *n* = 7), but the difference was not significant (*p* = 0.108). Striatal levels of total full-length TrkB and DARPP-32 remain unchanged with 0.1 mg/kg fingolimod treatment in 4-week-old R6/2 mice (Figure 3c,d). The ratio of activated phospho-TrkB–total TrkB in the striatum is significantly lower in fingolimod-treated 4-week-old R6/2 mice (fingolimod: 0.88 ± 0.09, *n* = 8; saline: 1.10 ± 0.04, *n* = 7; *p* < 0.050) (Figure 3f).

**Figure 4:**
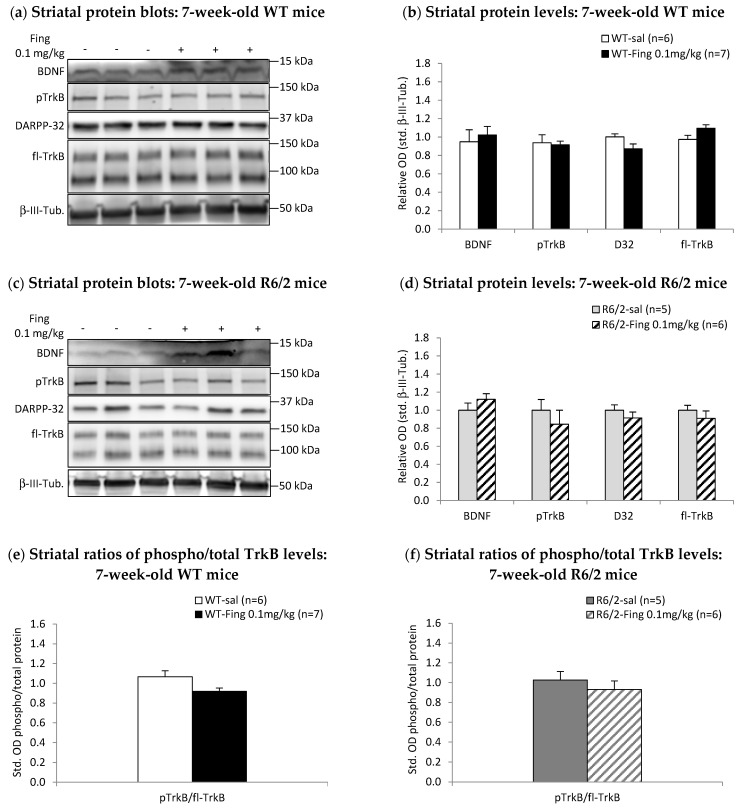
Effects of acute fingolimod treatment on BDNF-TrkB signaling proteins in 7-week-old WT and R6/2 mice. At 48 h after a single injection of saline (sal) or 0.1 mg/kg fingolimod (Fing), striatal tissues were extracted and analyzed by Western blot. (**a**) Representative blots of striatal proteins (rows) from individual (columns) WT mice treated at 7 weeks with saline (−) or fingolimod (+). (**b**) Graph of relative levels of striatal BDNF and downstream signaling proteins in WT mice normalized to saline controls. There are no significant differences between this low dose of fingolimod vs. saline treatment at 7 weeks. (**c**) Representative blots of striatal proteins from individual R6/2 mice treated at 7 weeks with saline (−) or fingolimod (+). (**d**) Graph of relative levels of striatal proteins in R6/2 mice shows no significant differences between this low dose of fingolimod vs. saline treatment at 7 weeks old. (**e**) Ratios of pTrkB/total TrkB levels in the striatum of 7-week-old WT mice and (**f**) R6/2 mice show that activation of TrkB signaling is not significantly different after fingolimod vs. saline treatment in WT mice and R6/2 mice.

To determine if the acute effects of 0.1 mg/kg fingolimod are distinct at later stages of HD, levels of BDNF and downstream signaling proteins were examined in 7-week-old R6/2 mice, a timepoint when overt motor symptoms are apparent in this HD model [52,61]. Striatal levels of BDNF and downstream proteins are similar between fingolimod-treated and saline-treated WT mice at 7 weeks old (Figure 4a,b). The ratio of phospho-TrkB–total TrkB in the striatum is similar between fingolimod- and saline-treated WT mice (Figure 4e). Moreover, 7-week-old R6/2 mice show no differences in striatal levels of BDNF and downstream proteins after acute fingolimod treatment compared to saline treatment (Figure 4c,d). The ratio of phospho-TrkB–total TrkB is similar in fingolimod- and saline-treated R6/2 mice (Figure 4f). Thus, in contrast to 4-week-old R6/2 mice that show a reduction in striatal BDNF levels following acute treatment with 0.1 mg/kg fingolimod, 7-week-old R6/2 mice show no change in striatal BDNF levels with this dose of fingolimod.

### 2.4. Fingolimod Dose Effect on BDNF-TrkB Signaling Proteins in Striatum of WT and R6/2 Mice

Since acute administration of 0.1 mg/kg fingolimod (above) revealed different trends in the BDNF-TrkB signaling pathway at early and later disease stages, further dose escalation was tested on additional cohorts of WT and R6/2 mice at age 4 or 7 weeks. Treatment groups received a single injection of either saline, 1.0 or 3.0 mg/kg of fingolimod, then 48 h later striatal tissue samples were extracted and assayed by Western blot. Motor cortex samples were also assayed, as this region is a major source of afferent BDNF for the striatum.

**Figure 5:**
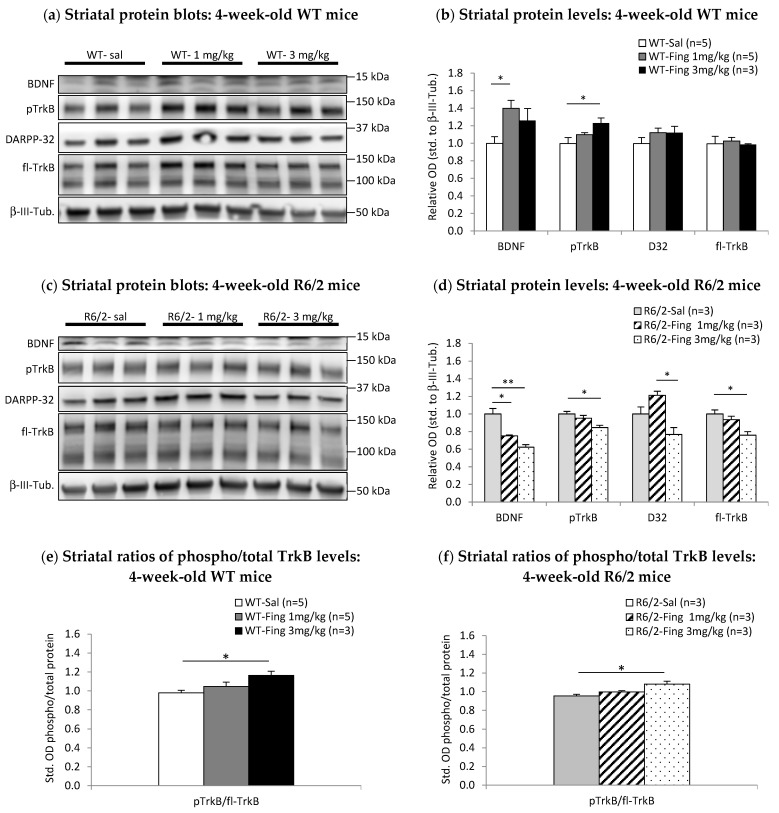
Acute effects of higher doses of fingolimod on BDNF-TrkB signaling proteins in 4-week-old WT and R6/2 mice. At 48 h after a single injection of either saline (Sal) or 1 mg/kg or 3 mg/kg fingolimod (Fing), striatal tissues were extracted and analyzed by Western blot. (**a**) Representative blots of striatal proteins (rows) from individual (columns) 4-week-old WT mice. (**b**) Graph of relative levels of striatal BDNF and downstream signaling proteins in WT mice normalized to saline controls. BDNF and pTrkB levels are higher after fingolimod vs. saline treatment at 4 weeks (* *p* < 0.050). (**c**) Representative blots of striatal proteins from individual 4-week-old R6/2 mice. (**d**) Graph of relative levels of striatal proteins in R6/2 mice shows that BDNF, pTrkB, and DARPP-32 are lower after fingolimod vs. saline treatment at 4 weeks old (* *p* < 0.050; ** *p* < 0.001). (**e**) Ratios of pTrkB/total TrkB levels in the striatum of 4-week-old WT mice and (**f**) R6/2 mice show that activation of TrkB signaling is elevated by fingolimod treatment in both WT mice and R6/2 mice (* *p* < 0.050 vs. saline).

In 4-week-old WT mice (Figure 5a,b), there is a significant main effect of fingolimod treatment on striatal levels of BDNF (F_2,12_ = 5.145; *p* < 0.050) and phospho-TrkB (F_2,12_ = 4.216; *p* < 0.050). Post hoc (LSD) comparisons show striatal BDNF levels are significantly higher in the 1.0 mg/kg dose group (rel. OD = 1.40 ± 0.09, *n* = 5; *p* < 0.050) compared to saline controls (rel. OD = 0.99 ± 0.08, *n* = 5) (Figure 5b). There is no further increase in BDNF levels in the 3.0 mg/kg group (rel. OD = 1.26 ± 0.14, *n* = 3; *p* = 0.354) compared to the 1.0 mg/kg group. Striatal phospho-TrkB levels are not significantly higher in the 1.0 mg/kg group (rel. OD = 1.10 ± 0.02, *n* = 5; *p* = 0.159) compared to saline controls (rel. OD = 1.00 ± 0.07, *n* = 5), but they are significantly higher in the 3.0 mg/kg group (rel. OD = 1.23 ± 0.06, *n* = 3; *p* < 0.050). Striatal levels of DARPP-32 and total full-length TrkB are unchanged by these higher doses of fingolimod. There is a significant main effect of fingolimod (F_2,12_ = 4.793; *p* < 0.050) on the ratio of activated phospho-TrkB–total TrkB in the striatum of 4-week-old WT mice (Figure 5e). This ratio is significantly higher than the 3.0 mg/kg group (1.40 ± 0.04, *n* = 3) compared to saline controls (0.98 ± 0.03, *n* = 5; *p* < 0.050) and marginally higher compared to the 1.0 mg/kg group (1.05 ± 0.02, *n* = 5; *p* = 0.080) (Figure 5e). Thus, in 4-week-old WT mice, acute administration of fingolimod increases striatal levels of BDNF and downstream activated phospho-TrkB receptors.

In cortical tissue from 4-week-old WT mice (Supplementary Figure S1a,b), there is a significant main effect of fingolimod (F_2,16_ = 3.766; *p* < 0.050) on BDNF levels. Post hoc comparisons show that cortical BDNF levels in the 3.0 mg/kg dose group (rel. OD = 3.31 ± 1.00, *n* = 6) are significantly higher compared to saline controls (rel. OD = 0.90 ± 0.22, *n* = 6; *p* < 0.050) and marginally higher compared to the 1.0 mg/kg dose group (rel. OD = 1.42 ± 1.00, *n* = 5; *p* = 0.070) (Supplementary Figure S1b).

In 4-week-old R6/2 mice (Figure 5c,d), there is a significant main effect of fingolimod on striatal levels of BDNF (F_2,8_ = 22.595; *p* < 0.050), phospho-TrkB (F_2,8_ = 6.979; *p* < 0.050), total full-length TrkB (F_2,8_ = 8.427; *p* < 0.050), and DARPP-32 (F_2,8_ = 10.378; *p* < 0.050). Post hoc (LSD) comparisons show that striatal BDNF levels are significantly lower in both 1.0 mg/kg (rel. OD = 0.75 ± 0.01, *n* = 3; *p* < 0.050) and 3.0 mg/kg (rel. OD = 0.62 ± 0.03, *n* = 3; *p* < 0.001) dose groups compared to saline controls (rel. OD = 1.00 ± 0.06; *n* = 3) (Figure 5d). phospho-TrkB levels are similar between saline controls (rel. OD = 1.00 ± 0.03, *n* = 3) and the 1.0 mg/kg group (rel. OD = 0.95 ± 0.03, *n* = 3), but they are significantly lower in the 3.0 mg/kg group (rel. OD = 0.84 ± 0.03, *n* = 3; *p* < 0.050). Full-length TrkB levels are significantly lower in the 3.0 mg/kg group (rel. OD = 0.76 ± 0.04, *n* = 3; *p* < 0.050) compared to saline controls (rel. OD = 1.00 ± 0.05, *n* = 3). DARPP-32 levels are similar between saline controls (rel. OD = 1.00 ± 0.08, *n* = 3) and the 1.0 mg/kg group (rel. OD = 1.21 ± 0.04, *n* = 3), but they are significantly lower in the 3.0 mg/kg group (rel. OD = 0.77 ± 0.08, *n* = 3; *p* < 0.050) compared to the 1.0 mg/kg group. There is a significant main effect of fingolimod (F_2,6_ = 8.263; *p* < 0.050) on the ratio of activated phospho-TrkB–total TrkB in the striatum of 4-week-old R6/2 mice (Figure 5f). This ratio is significantly higher in the 3.0 mg/kg dose group (1.08 ± 0.06, *n* = 3) compared to saline controls (0.95 ± 0.03, *n* = 3; *p* < 0.050). Thus, in contrast to WT mice, administration of fingolimod in 4-week-old R6/2 mice decreases striatal levels of BDNF and its receptor TrkB, ultimately diminishing downstream phospho-TrkB and DARPP-32 levels in the striatum.

In cortical tissue from 4-week-old R6/2 mice (Supplementary Figure S1c,d), there is a significant main effect of fingolimod treatment on BDNF levels (*F*_2,13_ = 8.272; *p* < 0.050). Post hoc comparisons show that cortical BDNF levels are significantly lower in the 3.0 mg/kg dose group (rel. OD = 0.62 ± 0.12, *n* = 5; *p* < 0.050) compared to the 1.0 mg/kg group (0.95 ± 0.06, *n* = 4; *p* < 0.050) and compared to saline controls (rel. OD = 1.03 ± 0.03, *n* = 5; *p* < 0.050) (Supplementary Figure S1d).

**Figure 6:**
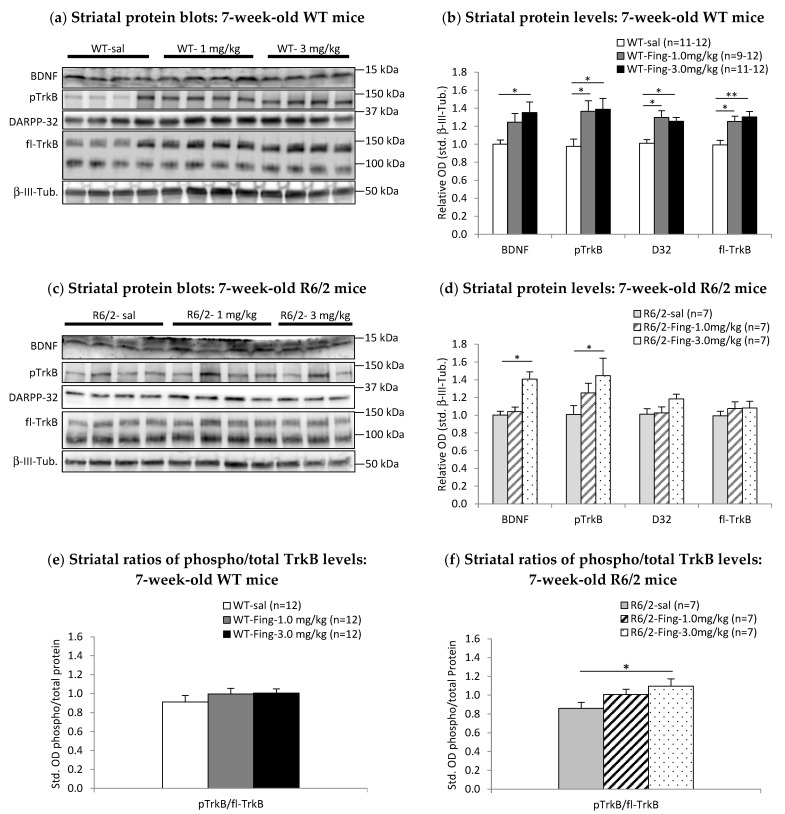
Acute effects of higher doses of fingolimod on BDNF-TrkB signaling proteins in 7-week-old WT or R6/2 mice. At 48 h after a single injection of either saline (Sal) or 1 mg/kg or 3 mg/kg fingolimod (Fing), striatal tissues were extracted and analyzed by Western blot. (**a**) Representative blots of striatal proteins (rows) from individual (columns) 7-week-old WT mice. (**b**) Graph of relative levels of striatal BDNF and downstream signaling proteins in WT mice normalized to saline controls. BDNF, pTrkB, DARPP-32, and fl-TrkB levels are higher after fingolimod vs. saline treatment at 7 weeks old (* *p* < 0.050, ** *p* < 0.001). (**c**) Representative blots of striatal proteins from individual 7-week-old R6/2 mice. (**d**) Graph of relative levels of striatal proteins in R6/2 mice shows that BDNF and DARPP-32 are higher after fingolimod vs. saline treatment at 7 weeks old (* *p* < 0.050 ). (**e**) Ratios of pTrkB/total TrkB levels in the striatum of 7-week-old WT mice and (**f**) R6/2 mice show activation of TrkB signaling is elevated by fingolimod treatment in R6/2 mice (* *p* < 0.050 vs. saline).

In 7-week-old WT mice, there is a significant main effect of fingolimod treatment on striatal levels of BDNF (F_2,30_ = 4.074; *p* < 0.050), phospho-TrkB (F_2,35_ = 4.575; *p* < 0.050), DARPP-32 (F_2,35_ = 7.962; *p* < 0.050), and full-length TrkB (F_2,35_ = 8.680; *p* < 0.001) (Figure 6a,b). Post hoc (LSD) comparisons show that striatal BDNF levels are significantly higher in both 1.0 mg/kg (rel. OD = 1.30 ± 0.11, *n* = 9; *p* < 0.050) and 3.0 mg/kg (rel. OD = 1.35 ± 0.12, *n* = 11; *p* < 0.050) groups compared to saline controls (rel. OD = 1.00 ± 0.05, *n* = 11) (Figure 6b). Phospho-TrkB levels are significantly higher in both 1.0 mg/kg (rel. OD = 1.37 ± 0.12, *n* = 12; *p* < 0.050) and 3.0 mg/kg (rel. OD = 1.39 ± 0.12, *n* = 12; *p* < 0.050) groups compared to saline controls (rel. OD = 0.98 ± 0.08, *n* = 12). DARPP-32 levels are significantly higher in both 1.0 mg/kg (rel. OD = 1.30 ± 0.07, *n* = 12; *p* < 0.050) and 3.0 mg/kg (rel. OD = 1.26 ± 0.04, *n* = 12; *p* < 0.050) groups compared to saline (rel. OD = 1.01 ± 0.04, *n* = 12). Full-length TrkB levels are significantly higher in both 1.0 mg/kg (rel. OD = 1.25 ± 0.06, *n* = 12; *p* < 0.050) and 3.0 mg/kg (rel. OD = 1.30 ± 0.06, *n* = 12; *p* < 0.050) groups compared to saline controls (rel. OD = 0.99 ± 0.05, *n* = 12). There is no main effect of fingolimod (F_2,35_ = 0.798) on the ratio of phospho-TrkB–total TrkB in the striatum of 7-week-old WT mice. Phospho-TrkB–total TrkB ratios are similar between treatment groups (Figure 6e). Thus, in 7-week-old WT mice, acute fingolimod treatment increases striatal levels of BDNF and its receptor TrkB and also increases downstream activated phospho-TrkB and DARPP-32 levels.

In cortical tissue of 7-week-old WT mice (Supplementary Figure S2a,b), there is a marginally significant main effect of fingolimod (F_2,17_ = 2.944; *p* = 0.083) on BDNF levels. Post hoc comparisons show that cortical BDNF levels are significantly higher in the 3.0 mg/kg group (rel. OD = 1.83 ± 0.27, *n* = 5; *p* < 0.050) compared to saline controls (rel. OD = 0.99 ± 0.11, *n* = 6) (Supplementary Figure S2b).

In 7-week-old R6/2 mice, there is a significant main effect of fingolimod treatment on striatal levels of BDNF (F_2,20_ = 13.537; *p* < 0.001) (Figure 6c,d). Post hoc (LSD) comparisons show that striatal BDNF levels are significantly higher in the 3.0 mg/kg dose group (rel. OD = 1.41 ± 0.08, *n* = 7; *p* < 0.001) compared to saline controls (rel. OD = 1.00 ± 0.04, *n* = 7). The main effect of fingolimod treatment on striatal levels of phospho-TrkB (F_2,20_ = 2.413; *p* = 0.118) and DARPP-32 (F_2,20_ = 2.418; *p* = 0.117) did not reach statistical significance in 7-week-old R6/2 mice. However, post hoc comparisons show that phospho-TrkB levels are significantly higher in the 3.0 mg/kg group (rel. OD = 1.45 ± 0.20, *n* = 7; *p* < 0.050) compared to saline controls (rel. OD = 1.00 ± 0.10, *n* = 7), while DARPP-32 levels are marginally higher in the 3.0 mg/kg group (rel. OD = 1.18 ± 0.05, *n* = 7; *p* = 0.062) compared to saline controls (rel. OD = 1.01 ± 0.06, *n* = 7). There is a marginally significant main effect of fingolimod (F_2,20_ = 3.227; *p* = 0.063) on the ratio of phospho-TrkB–total TrkB in the striatum of 7-week-old R6/2 mice. This ratio is significantly higher in the 3.0 mg/kg group (1.10 ± 0.08, *n* = 7; *p* < 0.050) compared to saline controls (0.86 ± 0.06, *n* = 7) (Figure 6f). Thus, similarly to WT mice, acute administration of fingolimod in 7-week-old R6/2 mice increases striatal levels of BDNF and downstream activated phospho-TrkB receptors.

In cortical tissue of 7-week-old R6/2 mice (Supplementary Figure S2c,d), the main effect of fingolimod (F_2,11_ = 1.312) on BDNF levels is not statistically significant. Cortical BDNF levels tend to be higher in both 1.0 mg/kg (rel. OD = 1.18 ± 0.20, *n* = 5; *p* = 0.580) and the 3.0 mg/kg (rel. OD = 1.51 ± 0.28, *n* = 5; *p* = 0.143) dose groups compared to saline controls (rel. OD = 1.00 ± 0.15, *n* = 4), but the differences are not statistically significant in 7-week-old R6/2 mice (Supplementary Figure S2d).

## 3. Discussion

In HD patients, mutant huntingtin protein is expressed from fetal stages; however, clinical locomotor symptoms and pathological neuronal degeneration manifest mainly in adulthood [17,29,62]. This suggests a significant prodromal period when neuroprotective strategies may be applied. A variety of cell autonomous and non-autonomous mechanisms have been proposed for the prominent striatal neuron degeneration in HD [63,64], including excitotoxicity [62,65,66] and loss of afferent neurotrophic support [67,68,69]. We determined whether fingolimod, a drug known to modulate neurotrophin levels in the CNS, can augment BDNF levels and exert a protective effect in the R6/2 mouse model of HD, particularly when treatment is initiated at presymptomatic stages prior to the onset of motor symptoms and striatal neuronal degeneration.

Fingolimod, an analogue of the endogenous lipid signaling molecule sphingosine, is a drug with pleiotropic effects that modulates sphingosine-1-phosphate (S1P) receptor signaling in diverse cell populations [48,70,71,72]. It is used for therapy in Multiple Sclerosis, where its main mechanism of action is attributed to S1P receptor-mediated suppression of inflammatory activity in the periphery and in the CNS [37]. It also acts on neuronal S1P receptors, which can modulate glutamate release and excitatory activity [40,73,74,75,76], ultimately regulating activity-induced BDNF expression [49,77]. Impaired BDNF-TrkB neurotrophic signaling is an important mechanism underlying striatal degeneration in the R6/2 mouse model of HD [27,52,53,55]. We investigated whether fingolimod treatment in R6/2 mice, initiated at early stages before the onset of striatal neurodegeneration and motor symptoms, could upregulate BDNF expression and protect against progressive locomotor dysfunction. Our results indicate that initiating chronic fingolimod treatment in presymptomatic R6/2 mice fails to rescue them from deteriorating motor function. Moreover, exposing presymptomatic 4-week-old R6/2 mice to a single dose of fingolimod decreases cortical and striatal BDNF levels, attenuates downstream TrkB signaling, and reduces DARPP-32 expression, a phenotypic marker of mature striatal neurons [6,27,78]. In contrast, acute administration of fingolimod in symptomatic 7-week-old R6/2 mice increases cortical and striatal BDNF levels, an effect also seen in WT mice. Therefore, fingolimod’s effects on forebrain neurotrophin levels depend on disease state in this animal model of HD.

Previous work in different models of neurodegenerative disease suggests that fingolimod can improve motor and behavioral symptoms [72,79]. For example, the MeCP2 mutant mouse model of Rett’s syndrome shows patterns of progressive motor dysfunction similar to R6/2 mice [52], including progressive deterioration of spontaneous locomotion and motor coordination, dystonic limb clasping, and striatal degeneration over a 12-week lifespan [80]. Chronic treatment with fingolimod (0.1 mg/kg, i.p., every 4 days over 4 weeks) improves motor symptoms in MeCP2 mutant mice and increases forebrain BDNF levels [32]. Using a similar dosing regimen initiated in R6/2 mice during the presymptomatic phase (0.1 mg/kg, i.p., every 3.5 days from age 4 to 11 weeks), we show a lack of therapeutic effect of fingolimod on the severity of major motor symptoms, including spontaneous locomotor activity and motor coordination, and no effect on weight loss. Moreover, chronic treatment with fingolimod initiated in young presymptomatic R6/2 mice worsens limb clasping, a dystonic motor symptom that is reliably noted as R6/2 mice progress through their 13- to 15-week lifespan [52,61,81]. These results suggest that young R6/2 mice have distinct responses to fingolimod compared to MeCP2 mutant mice. MeCP2 mutant mice show pathologically decreased cortical neuron excitability from a young age [82,83], and enhanced excitatory activity by fingolimod provides a salutary effect [32]. In contrast, although young presymptomatic R6/2 mice exhibit relatively normal cortical neuron activity, aberrant receptor properties in cortical and striatal neurons render them vulnerable to adverse excitatory signaling [66,84,85,86,87]. This vulnerability may be exacerbated by fingolimod’s enhancement of neuronal glutamate release and excitatory activation. At symptomatic R6/2 stages, striatal neurons exhibit deficits in afferent glutamatergic signaling [86,88,89,90]. Therefore, at this later stage, fingolimod may exert therapeutic effects via enhanced excitatory transmission and neurotrophin signaling. Indeed, a previous study using R6/2 mice demonstrated that chronic fingolimod treatment initiated at symptomatic stages improves motor symptoms, elevates striatal BDNF levels, and promotes neurotrophic support for striatal neurons [35]. While the present study also finds that BDNF levels are elevated after chronic fingolimod treatment from presymptomatic through to symptomatic stages, initiation of treatment at presymptomatic stages ultimately fails to improve R6/2 motor symptoms.

Deficiencies in BDNF supply [22,53] or impaired TrkB receptor-mediated signaling [26,27] are important mechanisms that contribute to progressive striatal neuron degeneration and declining motor function in HD [23,25,91]. Increasing BDNF supply to the striatum may therefore be neuroprotective in HD. Previous studies show fingolimod can promote excitatory activity-associated gene transcription, including the expression of BDNF [32,76,92]. In wild-type cortical cultures, fingolimod can induce BDNF mRNA and protein expression in a manner dependent on excitatory activity via glutamate receptors [32,49]. Moreover, acute administration of fingolimod in WT mice promotes Erk1/2-CREB signaling and elevates cortical BDNF expression [32]. CREB signaling is a well-established pathway that underlies glutamatergic activity-induced BDNF expression [93,94]. The present results further demonstrate that fingolimod treatment increases cerebral cortical BDNF protein levels and promotes BDNF-TrkB signaling at the striatal target in both 4 and 7-week-old WT mice. In contrast, similar treatment in presymptomatic 4-week-old R6/2 mice decreases cortical BDNF levels, attenuates striatal BDNF-TrkB signaling, and decreases striatal DARPP-32 levels. We propose that this early impairment of striatal neurotrophic support occurs with chronic fingolimod in presymptomatic R6/2 mice, ultimately contributing to striatal pathology and exacerbating locomotor decline. Indeed, we found that initiating chronic fingolimod therapy in presymptomatic R6/2 mice fails to improve motor deficits and even worsens limb-clasping motor behavior.

Previous work suggests that acute administration of fingolimod (0.1 mg/kg) augments BDNF levels in both the cortex and striatum of WT mice when assayed by ELISA [32], a method that does not distinguish between different protein forms of BDNF, including pre-proBDNF (32 kDa), proBDNF (28 kDa), and mature BDNF (14 kDa) [95]. Other ELISA-based studies report no effect of sub-chronic or chronic fingolimod treatment on cortical or striatal BDNF levels in WT mice [33,58,96,97]. Using Western blot, we demonstrate that fingolimod treatment in 4- and 7-week-old WT mice mediates a positive drug-effect that elevates striatal levels of mature BDNF and increases downstream activated phospho-TrkB receptors. In contrast, in presymptomatic 4-week-old R6/2 mice, fingolimod (0.1 to 3.0 mg/kg) significantly decreases striatal BDNF levels. This reduction is accompanied by a parallel decline in downstream activated phospho-TrkB receptors and striatal DARPP-32 protein levels. Conversely, in 7-week-old symptomatic R6/2 mice, fingolimod increases striatal BDNF levels. This finding is consistent with prior reports of neurotrophic elevation following chronic treatment in symptomatic R6/2 mice [35] and other neurodegenerative models [32,33,58].

Accumulating evidence indicates that fingolimod enhances neuronal glutamate signaling and BDNF expression in several neurodegenerative models via S1P receptor-dependent mechanisms [32,33,58,76,92,98]. However, S1P receptor-independent pathways—such as epigenetic modulation via HDACs [99,100]—may also drive neurotrophic signaling and contribute to our observations. Despite the critical role of S1P receptors in neuronal development [101], their specific contribution to cellular dysfunction in HD remains unclear. While S1P receptor expression appears normal in symptomatic HD mice [102,103,104], our data suggest that S1P receptor function warrants further investigation across all stages of disease progression. 

Altered forebrain excitability may explain the differential responses to fingolimod across the R6/2 lifespan. Studies in R6/2 and other HD models indicate that prodromal changes, specifically a decreased ratio of synaptic to extrasynaptic NMDA receptors (NMDARs) [87,105,106] and impaired synaptic glutamate clearance [84,107,108], could increase neuronal vulnerability to excitotoxicity and disrupt neurotrophic signaling [106,109]. While synaptic NMDARs couple glutamate signaling to the Erk1/2-CREB pathway to drive BDNF expression [93], extrasynaptic NMDARs antagonize this pathway [110,111,112,113,114]. Consequently, in the cerebral cortex of presymptomatic R6/2 mice, fingolimod-induced glutamate release may cause spillover, preferentially activating extrasynaptic NMDARs and suppressing cortical and striatal BDNF levels. Other prodromal deficits, including reduced synaptic NMDAR and AMPAR currents and increased GABAergic inhibition [106,115], may further uncouple fingolimod-induced glutamate release from BDNF production [32,40,41,58]. Importantly, these cortical abnormalities reverse during the symptomatic phase, where synaptic NMDAR and AMPAR currents increase, and GABAergic inhibition decreases [106,115,116]. This shift likely facilitates the coupling of fingolimod-induced glutamate release to BDNF expression, allowing for salutary effects when the drug is initiated during the symptomatic phase. 

Beyond its direct effects on neurons, fingolimod may modulate forebrain neurotrophins by altering inflammatory pathways. Pro-inflammatory cytokines (e.g., TNFα, IL-1β, IL-6) are activated in R6/2 mice in a complex, stage-dependent manner, paralleling the progression of inflammatory pathology in human HD [117,118,119,120,121]. Presymptomatic HD patients and animal models of HD, including R6/2 mice, are characterized by comparatively low levels of pro-inflammatory cytokines and activated inflammatory cell populations [120,121,122]. In contrast, the symptomatic phase is marked by heightened peripheral inflammation, elevated CNS cytokine levels, and widespread astrocyte and microglial activation [120,121,122,123]. Consequently, fingolimod’s impact may depend on the prevailing inflammatory milieu. In pro-inflammatory states, fingolimod can dampen CNS inflammation by inhibiting NF-κB signaling and glial activation [124], thereby enhancing BDNF expression [38,103]. However, in the quiescent presymptomatic state, fingolimod may promote gliosis and cytokine production, leading to BDNF downregulation [125,126,127]. This effect may be amplified by the progressive decline of S1P, the endogenous ligand for S1P receptors [128,129]. The relative absence of endogenous S1P reduces competition for the receptor, potentially enhancing fingolimod’s agonist activity and its modulation of neuronal excitability and inflammation. Finally, given that HD alters liver metabolism [56,130] and blood–brain barrier permeability [131,132], fingolimod bioavailability likely shifts as the disease advances. Therefore, a comparative analysis of fingolimod pharmacokinetics and pharmacodynamics across presymptomatic and symptomatic R6/2 stages represents a critical avenue for future research.

## 4. Materials and Methods

### 4.1. Animals

Animal procedures were in accordance with the Canadian Council on Animal Care guidelines for ethical use and welfare of animals in research, as administered by the McGill University Animal Care Committee. Female mice with ovarian transplants from R6/2 mice (C57Bl6/J background; Jackson Laboratory, Bar Harbor, MA, USA) carrying a mutant HTT exon-1 transgene with 120 ± 5 CAG repeats, were mated with wild-type (WT) males from the same genetic background to obtain WT and R6/2 offspring. Only F1 generation offspring were used to mitigate generational instability of CAG-repeat length, which could prolong symptom onset in R6/2 strains with >160 CAG-repeats [61,122]. Tail-tip samples from newborn pups were tested for the R6/2 transgene, and CAG repeat size (125 ± 2.18 repeats) was confirmed by PCR genotyping (Laragen Inc, Culver City, CA, USA). WT and R6/2 littermates of both genders were used for chronic treatment experiments assessing behavioral performance and forebrain protein levels and for acute treatment experiments assessing forebrain protein levels. 

### 4.2. Drug Treatment

A cohort of WT and R6/2 littermates was divided into two chronic treatment groups receiving either fingolimod (0.1 mg/kg, i.p., Cayman Chemicals, Ann Arbor, MI, USA, #10006292; WT-Fing, *n* = 8; R6/2-Fing, *n* = 12) or saline (WT-sal, *n* = 8; R6/2-sal, *n* = 11) every 3.5 days (q3.5d) from postnatal day (P) 28 to 73 (10.5 weeks). Mice were placed in an open field apparatus at ages 9 and 11 to evaluate spontaneous locomotion. Limb clasping was evaluated at 6, 8, and 10 weeks. Rotorod performance was evaluated at 9 weeks. Finally, animals were sacrificed at 11 weeks for Western blot assays of forebrain protein levels. 

A second cohort of 4-week-old WT and R6/2 littermates was divided into two acute treatment groups receiving a single injection (i.p.) of either saline (WT-sal, *n* = 12; R6/2-sal, *n* = 7) or fingolimod (0.1 mg/kg; WT-Fing 0.1 mg/kg, *n* = 10; R6/2-Fing 0.1 mg/kg, *n* = 8) and then sacrificed 48 h later for Western blot assays. A third cohort of 4-week-old WT and R6/2 littermates was divided into three acute treatment groups receiving a single injection of either saline (WT-sal, *n* = 5; R6/2-sal, *n* = 3) or fingolimod at 1.0 mg/kg (WT-Fing 1 mg/kg, *n* = 5; R6/2-Fing 1 mg/kg, *n* = 3) or 3.0 mg/kg (WT-Fing 3 mg/kg, *n* = 3; R6/2-Fing 3 mg/kg, *n* = 3) and then sacrificed 48 h later for Western blot assays. 

A fourth cohort of 7-week-old WT and R6/2 littermates received a single injection of either saline (WT-sal, *n* = 12; R6/2-sal, *n* = 7) or fingolimod (0.1 mg/kg; WT-Fing 0.1 mg/kg, *n* = 10; R6/2-Fing 0.1 mg/kg, *n* = 8), and then, they were sacrificed 48 h later for Western blot assays. Finally, a fifth cohort of 7-week-old WT and R6/2 littermates was divided into three acute treatment groups receiving a single injection of either saline (WT-sal, *n* = 12; R6/2-sal, *n* = 7) or fingolimod at 1.0 mg/kg (WT-Fing 1 mg/kg, *n* = 12; R6/2-Fing 1 mg/kg, *n* = 7) or 3.0 mg/kg (WT-Fing 3 mg/kg, *n* = 12; R6/2-Fing 3 mg/kg, *n* = 7), and then, they were sacrificed 48 h later for Western blot assays.

### 4.3. Tissue Preparation and Western Blot Assays

Mice were sacrificed by guillotine decapitation, and whole brains were dissected, cooled in isopentane (−80 °C), and stored in a −80 °C freezer. Next, whole brains were mounted on a freezing microtome with Tissue Tek gel (Sakura Finetek, Torrance, CA, USA). Tissue samples were obtained from the primary motor cortex and the dorsolateral motor striatum using a 1.0 × 1.5 mm cylindrical micro-punch (Stoelting, Wood Dale, IL, USA). Tissue samples were lysed in 60 µL of RIPA buffer (containing protease and phosphatase inhibitors) and then centrifuged to obtain soluble protein fractions that were individually stored at −80 °C.

Tissue samples from either WT or R6/2 mice were processed on different Western Blots. Equal volumes of tissue lysates from individual mice were loaded in parallel lanes on 4–15% gradient gels, along with a lane of molecular weight protein markers (Kaleidoscope™ Prestained Protein Standards, BioRad, Hercules, CA, USA). Proteins were separated through SDS-PAGE and then transferred to Nitrosecellulose membranes. Protein blots were cut into horizontal sections delimited by molecular weight markers and blocked with 2.5% BSA-TBST or 5% milk-TBST. Blot sections were separately immuno-labeled with antibodies to detect mature BDNF at 14 kDa (#sc546, 1:500; Santa Cruz, Santa Cruz, CA, USA), phospho-TrkB at 145 kDa (#sc135645, 1:500; Santa Cruz), phospho-Erk1/2 at 42–43 kDa (#9106, 1:2000; Cell Signaling Tech., Danvers, MA, USA), DARPP-32 at 32 kDa (#ab1656, 1:2000; Millipore, Burlington, MA, USA), total full-length TrkB at 145 kDa (#5374, 1:2000; Millipore, USA), total Erk1/2 at 42–43 kDa (#9102, 1:2000; Cell Signaling Tech., USA), and β-III-Tubulin at 50 kDa (#PRB435, 1:10,000; Covance, Princeton, NJ, USA). Immuno-labeled blots were incubated in secondary HRP-conjugated antibodies (goat anti-rabbit or -mouse; 1:10,000; Millipore) and then developed using either Super Signal West Femto (Pierce, Appleton, WI, USA) or Clarity (Bio-Rad, USA; for full-length TrkB and Erk1/2) chemiluminescence substrate and digitally imaged using a high-resolution (3-megapixel) and high-sensitivity (16-bit) camera system (Chemocam Imager, Intas Science Imaging, Göttingen, Germany).

Labimage 1D software (version L360, Kapelan Bio-Imaging Solutions, Leipzig, Germany) was used to quantify the optical density (OD) of specific protein bands. The OD value of each protein band was standardized to that of β-III-Tubulin (a neuronal-specific protein) in the same sample lane, thereby controlling for total neuronal protein loading for each individual tissue sample. The final relative OD value for each protein band was calculated relative to the average OD value of the saline-treated samples in each blot [133]. Each blot always included all treatment groups in order to calculate the relative OD value of each sample. Samples were run multiple times in each mouse to obtain at least 3 blots, and relative OD values were averaged for individual animals. Subsequently, individual values were averaged within each treatment group.

### 4.4. Evaluation of Motor Behavior

WT and R6/2 mice were weighed prior to receiving chronic drug treatments every 3.5 days from age P28 to P74. Locomotor activity was assessed between ages P63 and P77 by testing spontaneous ambulatory movement in an open field apparatus with infrared backlighting [134]. Spontaneous movement was recorded during a one-hour period using the Videotrack system equipped with motion analysis software (VideoTrack 3.1, Viewpoint Life Sciences, Montreal, Canada), and the total distance traveled was calculated.

Limb dystonia was assessed at ages P42, P56, and P70 by testing limb clasping during 20 s tail suspensions repeated three times with a 30 min rest in between each trial. Limb movements were videotaped and later scored by an observer blinded to genotype. Clasping movements were observed as a retraction of a limb toward the body. Clasping score was based on an index of clasps towards midline for each limb: none = 0, no clasping; mild = 0.25, limb retraction towards midline but contraction not sustained (<5 s); moderate = 0.5, high-amplitude limb retraction to or beyond the midline that was not sustained or partial limb retraction that was sustained (>5 consecutive seconds); severe = 0.75, high-amplitude limb retraction beyond the midline that was sustained (>5 s). Scores for each limb are added for a maximum total limb clasping score of 3. The highest clasping score from the 3 sessions was used for analysis [81].

Motor coordination was assessed at age P67 by testing performance on a Rotorod apparatus (Columbus Instruments, Columbus, OH, USA) during an accelerating rotation protocol [135,136]. Rotorod baseline training occurred over 3 consecutive days with four 5 min trials on each day. The maximum speed at the end of the training period was 24 r.p.m. Testing on day 4 consisted of five trials. At each trial, the speed was gradually increased from 5 to 44 r.p.m. over 5 min, with a 1-hour rest period between trials. The latency to fall at each trial was recorded, and the mean of the 3 best performances was used for analysis. 

### 4.5. Statistical Analysis

Motor behavior scores and weight for the fingolimod and saline groups were compared by two-way ANOVA for main effects and interaction of age and treatment. Post hoc comparisons were carried out to identify simple effects between age or treatment groups, as detailed in the results. Non-parametric statistics were used to analyze the results of clasping behavior. Protein levels are reported as mean relative optical density (rel. OD ± SEM) of groups based on genotype and drug treatment (saline, 0.1, 1.0, and 3.0 mg/kg). Relative protein levels were compared by one-way ANOVA with appropriate post hoc tests to identify differences between treatment groups.

## 5. Conclusions

Fingolimod, an approved immunomodulator for relapsing-remitting Multiple Sclerosis, exhibits complex and stage-dependent effects on neurotrophin pathways and motor behavior in Huntington’s disease mouse models. When initiated in the presymptomatic phase, fingolimod decreases BDNF levels and TrkB signaling in the forebrain and fails to improve motor behavior. However, during the symptomatic phase, acute fingolimod administration enhances striatal BDNF levels and TrkB receptor signaling, consistent with previous findings indicating its potential to improve motor behavior when administered during this later stage. Further research is warranted to elucidate the mechanisms underlying these stage-dependent effects, particularly the influence of the evolving excitability and inflammatory environments as Huntington’s disease progresses. These findings emphasize the importance of tailoring treatment approaches in HD and other neurodegenerative diseases, recognizing the dynamic nature of disease progression and the complex interactions between immunomodulatory drugs and neurotrophin signaling pathways.

## Data Availability

The raw data supporting the conclusions of this article will be made available by the authors on request.

## Supplementary Materials

The following supporting information can be downloaded at: https://www.mdpi.com/article/10.3390/ijms27010494/s1.

## Abbreviations

BDNF	Brain-derived neurotrophic factor
NMDA	N-methyl-D-aspartate
AMPA	α-amino-3-hydroxy-5-methyl-4-isoxazolepropionic acid
HD	Huntington’s disease
