# Supplementary material for: Fingolimod Effects on Motor Function and BDNF-TrkB Signaling in a Huntington’s Mouse Model Are Disease-Stage-Dependent
Source: Int J Mol Sci. 2026 Jan 3;27(1):494. doi: 10.3390/ijms27010494 (PMC12787111; doi:10.3390/ijms27010494)
Supplement: Supplementary file 1 [file ijms-27-00494-s001.zip › ijms-3451265-supplementary.pdf]

Supplemental Figures

(a) Cortical protein blots: 4-week-old WT mice

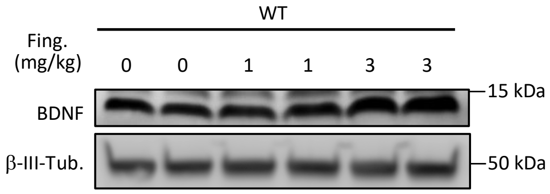

(b) Cortical protein levels: 4-week-old WT mice

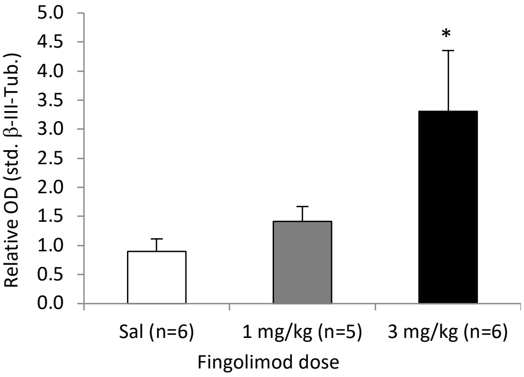

(c) Cortical protein blots: 4-week-old R6/2 mice

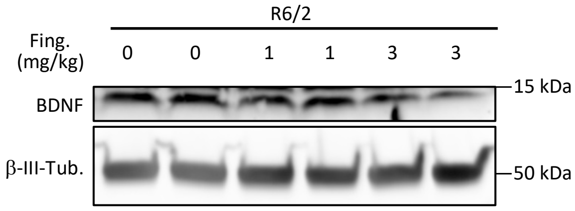

(d) Cortical protein levels: 4-week-old R6/2 mice

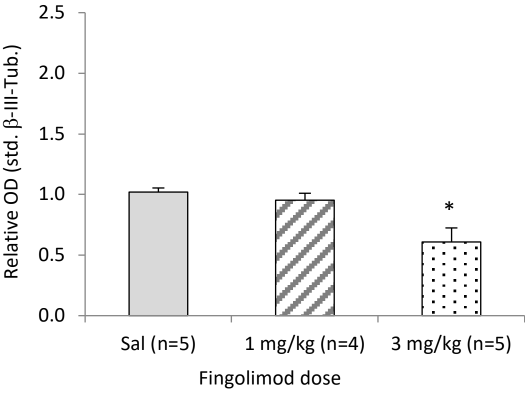

Figure S1. Acute effects of higher doses of fingolimod on 4-week-old WT and R6/2 mice. (a) Representative blot of cortical proteins (rows) from individual (columns) 4-week-old WT mice. (b) Graph of relative levels of BDNF protein in the motor cortex of 4-week-old WT mice. BDNF levels are significantly higher in fingolimod- (3 mg/kg) vs. saline-treated WT mice e) Representative blot of cortical proteins from individual 4-week-old R6/2 mice. f) Graph of relative levels of cortical BDNF protein in R6/2 mice shows a significant decrease in fingolimod (3 mg/kg) vs. saline treatment at 4-weeks-old (*p < .050 vs. saline)

(a) Cortical protein blots: 7-week-old WT mice

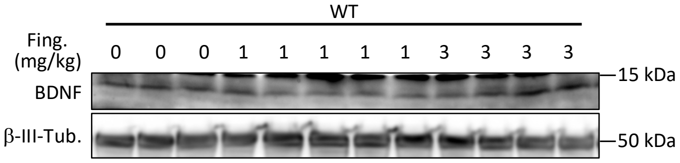

(b) Cortical protein levels: 7-week-old WT mice

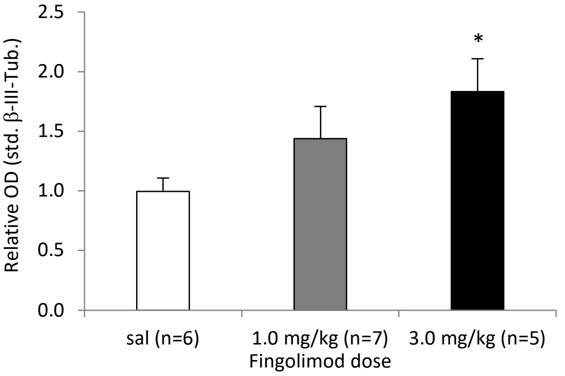

(c) Cortical protein blots: 7-week-old R6/2 mice

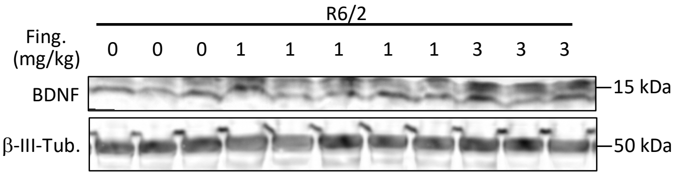

(d) Cortical protein levels: 7-week-old R6/2 mice

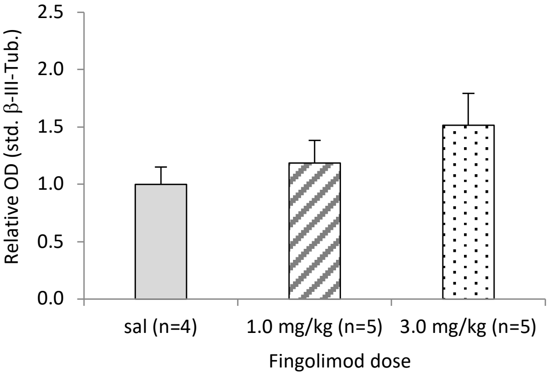

Figure S2. Acute effects of higher doses of fingolimod on 7-week-old WT and R6/2 mice. (a) Representative blot of cortical proteins (rows) from individual (columns) 7-week-old WT mice. (b) Graph of relative levels of BDNF protein in the motor cortex of 7-week-old WT mice. BDNF levels are significantly higher in fingolimod (3 mg/kg) vs. saline-treated WT mice. (c) Representative blot of cortical proteins from individual 7-week-old R6/2 mice. (d) Graph of relative levels of cortical BDNF protein in R6/2 mice shows a trend of higher levels with fingolimod treatment but the differences are not statistically significant.
